# Case of Fracture of Inferior Maxilla, Treated by a Wire Gripe

**Published:** 1863-08

**Authors:** A. J. Hyde

**Affiliations:** East Hardwick, Vt.


CASE OF FRACTURE OF INFERIOR MAXILLA, TREATED BY A WIRE GRIPE.—*By A. J. Hyde, M. D., of East Hardwick, Vt.*—Mr. F. Farrington, merchant, aged 25 years, received a kick from a horse, fracturing his lower jaw, between the bicuspids, on the right of the symphysis.

I saw him about two hours after the injury, in a half-conscious state, he having been severely stunned by the blow. The horse shoe had cut through the soft parts underneath the chin, the blow being directed upwards and backwards. Mobility was complete, the fragments moving half an inch upon each other without difficulty. The crepitation was distinct. Blood and saliva were flowing freely from the wound and mouth. Both teeth adjoining the fracture were loose, but I decided to attempt to save them both. Reaction came on; the concussion of the brain passed off in a day or two by the use of cathartics, and the only point before me was to keep the fracture in place. I was three days in trying many different appliances recommended by authors in case of this fracture. They each entirely failed to keep the portions of bone together. I really could find nothing in books which, being followed out, would answer the requirements of the case, for each time the patient swallowed the fracture would spread apart. I then hit upon the plan of gripping the teeth with wire. I simply wanted a pair of nippers, a dental file for separating the teeth, and a piece of fine wire. Of the latter I happened to find some the ladies use in making hair wreaths. The second tooth on either side of the fracture being firm, I filed down between that and the next, it being between the first and second molars on one side, and the bicuspid and incisor on the other, this being done without causing much pain. I then took five or six strands of the wire, untwisted, in a piece of sufficient length to pass round through these two places of separated teeth, thus embracing the fracture with it. It being tucked down as near the alveolar process as possible on each side, I brought the two ends of the wires together externally, took my nippers, and twisted them together until the fracture was brought perfectly in place. This was all the means I used, and I was gratified to see the portions of jaw remain firmly in place.

His food, of course, consisted wholly of liquids. The blood oozed slightly from the wound and mouth for five or six days, and then ceased. I only claim for this that it is simple, practicable, and efficient, where the fracture is between teeth.

I need hardly add that union in due time was perfect and the gripe was removed. The two loose teeth were saved, and are firm and good now, nearly four years after the accident.—*American Medical Times*, Aug. 29, 1863.

